# Ethical and Social Issues for Health Care Providers in the Intensive Care Unit during the Early Stages of the COVID-19 Pandemic in Japan: a Questionnaire Survey

**DOI:** 10.1007/s41649-021-00194-y

**Published:** 2021-11-15

**Authors:** Yusuke Seino, Yayoi Aizawa, Atsushi Kogetsu, Kazuto Kato

**Affiliations:** Department of Biomedical Ethics and Public Policy, Graduate School of Medicine, Osaka University, Suita, Osaka Japan

**Keywords:** COVID-19, Moral distress, Clinical ethics, Ethical issues, Social issues, Intensive care unit, Japan

## Abstract

This questionnaire-based observational study was conducted in July 2020 with the aim of understanding the ethical and social issues faced by health care providers (HCPs) registered with the Japanese Society of Intensive Care Medicine in intensive care units (ICUs) during the coronavirus disease (COVID-19) pandemic. There were 200 questionnaire respondents, and we analyzed the responses of 189 members who had been involved in COVID-19 treatment in ICUs. The ethical and social issues that HCPs recognized during the pandemic were difficulties in the decision-making process with patients’ families, limitations of life-sustaining treatment, lack of palliative care, and inadequate mental support for patients’ families and HCPs. Regarding decision-making on issues of clinical ethics during the pandemic, more than half of the respondents thought they had failed to provide sufficient palliative care to patients and responded that they experienced moral distress. The free-text responses on moral distress revealed issues such as unusual treatment and care, restricted visits, challenging situations for HCPs, and psychological burden. Additionally, 38.1% of respondents experienced episodes of social prejudice or discrimination and 4.7% experienced a shortage of medical resources. Our study result shows that the moral distress of HCPs was caused by difficulties in patient-centered decision-making and insufficient medical care to patients and their families. These were caused mainly by a lack of communication due to the stronger implementation of infection control measures. We believe that it is important to address ethical and social issues during a pandemic in order to provide appropriate medical care and prevent burnout among HCPs.

**Supplementary Information:**

The online version contains supplementary material available at 10.1007/s41649-021-00194-y.

## Background

The COVID-19 outbreak, which was first reported in China, advanced rapidly worldwide and was officially declared a pandemic by the World Health Organization on March 11, 2020 (Mannelli 2020; WHO 2020). During the initial phase of the pandemic, countries such as Italy (Mannelli 2020; Remuzzi and Remuzzi 2020) and the USA (Richardson et al. 2020) were unable to adequately respond to the increasing number of patients and the shortage of medical resources, including intensive care unit (ICU) beds and ventilators (Truog et al. 2020; Emanuel et al. 2020), which led to a humanitarian crisis in those regions (Nacoti et al. 2020).

In Japan, a state of emergency was declared for the first time on April 7, 2020, owing to the spread of COVID-19 mainly in urban areas. Infection control measures, such as refraining from going out, closing schools, testing for COVID-19, and isolating those who tested positive, were implemented. There was a significant impact on the healthcare delivery system, especially in the ICU. Although Japan’s healthcare system has a large number of general beds, the number of ICU beds is small (OECD 2020). Therefore, as the number of critically ill patients increases, the medical capacity in ICUs is likely to be exceeded (Japan Medical Association COVID-19 Expert Meeting 2020). During the initial phase of the pandemic, although the number of seriously ill patients was lower in Japan than in other countries, facilities accepting critically ill COVID-19 patients were limited because of a lack of adequate infection control measures and equipment shortages. Therefore, the burden on some facilities became increasingly severe.

Even at normal times in the ICU, HCPs encounter ethical and social issues, such as decision-making at the end of life and palliative care (Makino et al. 2014). Addressing these issues is essential because it will lead to the provision of desirable medical care to patients and their families. In addition, challenging ethical situations, exposure to high patient mortality, and difficult daily workloads can lead to excessive stress for HCPs in the ICU, leading to burnout syndrome (Kerlin et al. 2020). Especially, moral distress has been identified as one of the significant causes of burnout (Colville et al. 2019). It is thought to be caused by HCPs’ inability to provide appropriate treatment and care owing to institutional restrictions (Cacchione 2020; Corley 2002; Morley et al. 2020). HCP burnout has far-reaching and significant consequences, not only for the personal well-being of HCPs but also for patient care and the healthcare system (Kerlin et al. 2020). Therefore, it is important to identify the ethical and social issues in the ICU and to address the resulting moral distress and burnout.

Although it is likely that the changes in social conditions by the COVID-19 pandemic have made the ethical and social issues faced by HCPs in ICUs more apparent and serious, the actual experiences and perceptions of the HCPs are not clearly understood. In this study, we administered a questionnaire to HCPs regarding the ethical and social issues and moral distress they experienced before and during the pandemic.

## Objectives

The purpose of this study was to understand what ethical and social issues ICU HCPs face in Japan during the COVID-19 pandemic and suggest possible measures to deal with them.

## Methods

### Study Design and Participants

This was a cross-sectional, questionnaire-based observational study of registered Japanese Society of Intensive Care Medicine (JSICM) members who were contacted using the JSICM mailing list. The questionnaire was sent to the 10,767 members (7717 physicians, 2122 nurses, 484 clinical engineers, and 444 others), and the electronic survey was conducted anonymously using Google Forms. We found no publicly available data on the percentage of ICU HCPs who had been engaged in the COVID-19 treatment at the time of the survey, so we were unable to identify the demographics of the population. Initially, this study intended to include all members of JSICM. However, as there were few responses from members who had not been involved in the COVID-19 treatment, it was difficult to make statistical comparisons with those who were involved in the treatment of COVID-19. Hence, in this paper, we present only the results of the analysis of responses of members who have been involved in the treatment of COVID-19.

This study was approved by the Clinical Trial Group Committee of the JSICM and the Ethics Review Committee of Osaka University (Ethics Review No. 20095). Consent for the survey was obtained when a participant answered survey questions.

The survey was conducted over a 2-week period, between July 6 and 19, 2020, when the number of infected people in Japan had decreased and the state of emergency was lifted.

### Questionnaire

The survey questions pertained to (1) respondent demographics; (2) ethical and social issues encountered in the ICU during normal times (i.e., before the COVID-19 pandemic); (3) changes in the medical care system, ethical and social issues, and moral distress experienced in the ICU during the pandemic; and (4) allocation of medical resources during the pandemic. The questionnaire included 34 items in multiple-choice and free-text response formats (Table S1).

### Data Analysis

Quantitative data were analyzed using EZR statistical software (Saitama Medical Center, Jichi Medical University, Saitama, Japan) (Kanda 2013). The *χ*^2^ test was used to compare groups. *P* < 0.05 was considered statistically significant.

Qualitative data obtained from the free-text response was examined using inductive content analysis (Elo and Kyngäs 2008). One author (YS) assigned the text content of all qualitative data into categories and subcategories and coded the text in terms of content. When an individual’s response spanned multiple categories, it was classified into such. Two other authors (YA and KK) audited the original inductive content analysis and recategorized it, as necessary. Discrepancies were discussed until consensus was reached. The coding was agreed upon by all authors for consistency and validity.

## Results

### Respondent Characteristics

Two hundred respondents answered the questionnaire. Two duplicates and nine respondents who had not been involved in the treatment of patients with confirmed or suspected COVID-19 cases were excluded. In the end, 189 responses were analyzed. The respondents’ characteristics are summarized in Table 1. Only 7.4% of the respondents indicated that their institutions did not have a mechanism for examining clinical ethical issues.

**Table 1:** Characteristics of respondents (*N* = 189)

Variable	*N* (%)
**Profession**	
Physicians	97 (51.3%)
Nurses	65 (34.4%)
Clinical engineers	19 (10.0%)
Physical and occupational therapists	7 (3.7%)
Pharmacist	1 (0.5%)
**Years of ICU experience**	
No experience	1 (0.5%)
1–2 years	11 (5.8%)
3–5 years	19 (10.1%)
6–10 years	44 (23.2%)
11–20 years	83 (44.0%)
More than 21 years	31 (16.4%)
**Working experience in the intensive care unit**	
Currently working full-time in the intensive care unit	103 (54.5%)
Currently working concurrently in the intensive care unit	47 (24.9%)
Worked in the intensive care unit in the past and currently indirectly in intensive care unit work	26 (13.8%)
Worked in the intensive care unit in the past and not currently involved in intensive care unit work	12 (6.3%)
Has not worked in the intensive care unit in the past but currently involved indirectly in intensive care unit work	1 (0.5%)
**Type of intensive care unit before the coronavirus disease (COVID-19) pandemic**	
Open intensive care unit^a^	28 (14.8%)
Semi-closed intensive care unit^b^	107 (56.7%)
Closed intensive care unit^c^	54 (28.5%)
**Facilities**	
University hospital	90 (47.6%)
Public hospitals	54 (28.6%)
Other	45 (23.8%)
**Mechanisms for examining clinical ethical issues (multiple choice)**	
An independent hospital ethics committee	122 (64.6%)
Other ethics committees	41 (21.7%)
An ethics consultation system	34 (18.0%)
No mechanism for considering clinical ethical issues	14 (7.4%)

^a^Intensivists are the patient’s primary attending physician

^b^Mandatory critical care consultation (intensivists are not the patient’s primary attending physician, but every patient admitted to the intensive care unit receives a critical care consultation) or elective critical care consultation (intensivists are involved in the care of the patient only when the attending physician requests a consultation)

^c^No critical care physician (intensivists are unavailable)

### Ethical and Social Issues and Moral Distress in Japanese ICUs in Normal Times

Regarding the ethical and social issues they encountered in ICUs during normal times before the pandemic, the respondents’ answers were as follows: limitation of life-sustaining treatment (66.1%), difficulties in decision-making with the patient’s family (61.9%), with HCPs (55.6%), and with patients (43.4%) (multiple-choice question) (Fig S1).

The most common method for making decisions on clinical ethical issues among HCPs was multidisciplinary meetings (47.6%). Only 7.4% of respondents answered that decisions were made by a physician alone (Table S2).

Moral distress was routinely experienced by half of the respondents when working in the ICU. Moral distress was significantly more common among nurses than among physicians (*P* = 0.003) (Table 2).

**Table 2:** Number of health care providers who experienced moral distress in ICU care in normal times and during the coronavirus disease pandemic

	Overall (*N* = 189)	Physicians (*N* = 97)	Nurses (*N* = 65)	Others (*N* = 27)	
Normal times	107 (56.6%)	47 (48.4%)*	46 (70.8%)*	9 (33.3%)	*P = 0.003
During the pandemic	108 (56%)	45 (46.3%)*	49 (75.3%)*	14 (51.8%)	*P < 0.001

^*^χ^2^ tests

*ICU*, intensive care unit

### Ethical and Social Issues and Moral Distress in Japanese ICUs during the COVID-19 Pandemic

In this section, we examined the influence on the medical care system and the ethical and social issues experienced by HCPs during the COVID-19 pandemic.

**Fig. 1:**
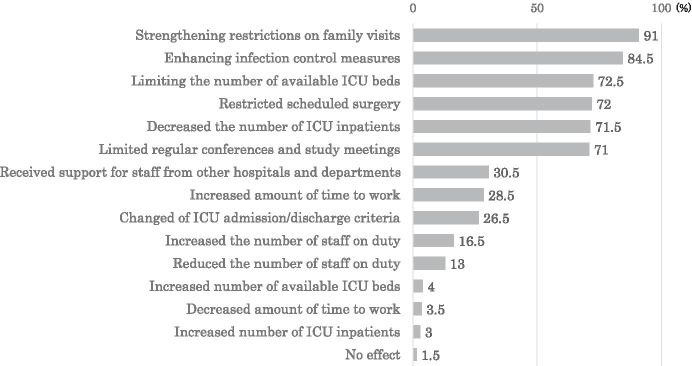
Impact of the coronavirus disease pandemic on the medical care system (multiple-choice question)

Respondents mentioned changes in the medical care system due to the COVID-19 pandemic in the following order: strengthening restrictions on family visits, enhancing infection control measures, limiting the number of available ICU beds, and restricting scheduled surgery (Fig. 1).

**Fig. 2:**
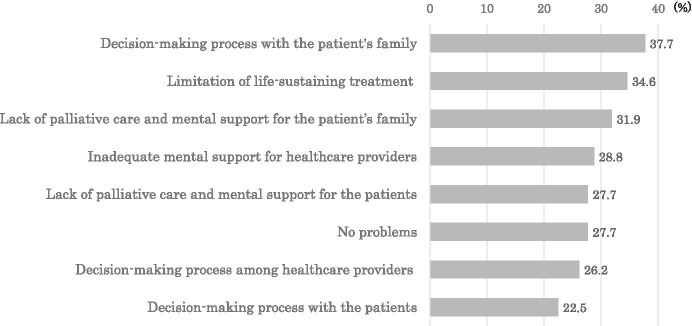
Ethical and social issues related to providing medical care recognized by health care providers more during the COVID-19 pandemic than in normal times (multiple-choice question)

The ethical and social issues faced while providing medical care that HCPs identified as being more problematic during the COVID-19 pandemic were difficulties in the decision-making process with patients’ families, limitations of life-sustaining treatment, lack of palliative care, inadequate mental support for the patients’ families, and mental support for HCPs (Fig. 2).

The decision-making method regarding clinical ethical issues among HCPs remained mostly unchanged during the COVID-19 pandemic (Table S2).

When making decisions in situations where clinical ethical issues arose during the COVID-19 pandemic, more than half of the respondents said they had failed to provide sufficient palliative care to patients as well as mental support and appropriate information to both patients and their families (Fig. S2). They also said that patients and their families could not speak openly with HCPs about their thoughts and concerns, and it was difficult for the HCPs to understand them. The main reasons for insufficient care, as described in the free-text responses, include visiting restrictions, increased implementation of infection control measures, anxiety about COVID-19, lack of communication between HCPs, patients, and their families, inability to confirm patients’ wishes, and the need for unusual care.

More than half of the HCPs experienced unusual moral distress during the pandemic. The proportion of HCPs experiencing unusual moral distress during a pandemic was significantly higher among nurses than among physicians (*P* < 0.001) (Table 2).

Seventy-six free-text responses on moral distress were classified into five categories (Table 3). The most common responses were unusual treatment and care such as limited contact with patients and deep sedation and muscle relaxation, to prevent infection among HCPs. Many respondents also answered that patients and their families are unable to meet owing to restricted visits.

**Table 3:** Description of moral distress during the COVID-19 pandemic (free-text response)

Category	Subcategory	Quotes
1. Unusual treatment and care		
	1) Limitation on medical treatment and care and deterioration in quality caused by prioritizing infection control	• Infection control based on hospital policy was prioritized over the rights of patients and medical providers • Deep sedation and muscle relaxation, which is not normally necessary, was used to prevent infection by health care providers • Because of concerns about the risk of infection to medical providers, contact with patients was restricted, and the usual treatment could not be performed
	2) Problems of medical treatment and care other than infection control	• There were situations where we had to give up, even when we would not normally give up • Support nurses from general wards who had little experience in caring for critically ill patients also provided care to these patients, resulting in differences in the quality of nursing • When there were too many patients, it was necessary to triage patients to get them admitted to the ICU • Insufficient medical resources
2. Restricted visits		
	1) Patient and family could not meet	• Patients who were encouraged by family visits could not see them due to visiting restrictions • No one was allowed to visit the patients before or after the patient died • There were restrictions on visits to patients who do not have COVID-19
	2) Difficulty in dealing with visitation restrictions	• It was difficult to establish a visitation system with infection control measures, both financially and in terms of information management; hence, restrictions on family visitations were not easily resolved • We wanted patients and families to meet online or exchange photos but could not because the hospital did not give approval
3. Challenging situations for health care providers		
	1) Conflict and prejudice	• There was conflict and a feeling of unfairness between staff who were involved in COVID-19 practice and those who were not • Low awareness of infection prevention among physicians and staff who were not involved in COVID-19 practice • The physician did not enter the room of the COVID-19 patient and gave instructions from outside the patient’s room. The nurses were doing most of the work in the room
	2) System of hospitals for COVID-19	• Opinions from people on the frontline were not communicated to hospital administrators • There was no physician to take leadership
	3) Support system for health care providers	• Harmful effects due to workload, fatigue of healthcare providers • Conflict between ensuring staff safety and accepting COVID-19
4. Psychological burden		
	1) Fear of infection	• I could not work because I was worried about my family • If I had an asymptomatic infection, I might infect my family or others. I was afraid that information about infected people would be reported and that the infection would spread
	2) Conflict in medical treatment and care	• It was hard for many staff members to maintain a sense of normality • I was not in a psychological state where I could make normal judgments. At the same time, I was working with a feeling of fatigue
5. Others		
		• Overall social activities were restricted to prevent infection in the elderly • Differences in perceptions between the public and health care providers lead to excessive fear and discriminatory behavior

Regarding whether they experienced social prejudice or were discriminated against as a HCP during the COVID-19 pandemic, 38.1% answered that they had experienced an episode of prejudice or discrimination. Among them, 19.6% experienced a mental burden. Meanwhile, 28.6% of the respondents said that, while they did not experience prejudice or discrimination, they hesitated to directly interact with others, and 31.7% said they did not experience prejudice or discrimination.

Regarding advanced preparations for the allocation of medical resources necessary for life support, 20.1% of the affiliated facilities prepared in-facility guidelines, and 50.3% of the respondents discussed necessary medical resource allocation beforehand. Of the respondents, 4.7% experienced a shortage of medical resources to the extent that necessary treatment and care could not be provided. On the contrary, 5.8% of the respondents were able to receive support, such as patient transfer to another facility; 18.5% were able to increase medical resources; and 24.9% were able to provide care with existing medical resources. For 43.9% of the respondents, the shortage of medical resources was not a concern. The medical resources (including medical personnel) that were in shortage included personal protective equipment (PPE) required for medical procedures (70.9%), nurses (45.0%), physicians (33.3%), and ICU beds (28.0%) (multiple-choice question).

## Discussion

This questionnaire-based study investigated the ethical and social issues encountered in Japanese ICUs and how these changed during the COVID-19 pandemic. To the best of our knowledge, this has not been previously reported. According to the results of this study, ICU HCPs became aware of various ethical and social issues and experienced moral distress during the COVID-19 pandemic.

Since there are no official statistics on the number of ICU HCPs involved in the treatment of COVID-19 patients in Japan, it is difficult to identify how many of the JSICM members were engaged in COVID-19 treatment at the time of the survey. However, according to the limited official statistical data, while the total number of ICU beds in Japan is about 7000, the maximum number of critically ill patients up until July 2020, the end point of the questionnaire, was only about 330 per day. Judging from this, we estimate that only a small number of HCPs in the ICU were involved in the treatment of COVID-19 patients. Therefore, despite the limited number of responses, we believe that the results of this survey are meaningful as they reflect the situation in the early stages of the COVID-19 pandemic in Japan.

### Ethical and Social Issues and Moral Distress in Japanese ICUs During the COVID-19 Pandemic

One of the impacts of the COVID-19 pandemic on Japanese ICUs was that visitation restrictions and increased implementation of infection control measures made it difficult for patients, their families, and HCPs to communicate with each other. Furthermore, because of the pandemic, it was necessary to provide treatment and care with unusual restrictions, and HCPs experienced moral distress from not being able to provide sufficient treatment to patients. HCPs also experienced discrimination against themselves, as well as shortages of medical resources. These ethical and social issues can be highly stressful for HCPs and may cause burnout.

#### Communication

In order to make patient-centered decisions, it is necessary to have opportunities to provide sufficient information to patients and their families and have discussions based thereon. However, during the COVID-19 pandemic, communication between HCPs, patients, and their families became difficult.

Communication with other HCPs is important so that they can discuss the medical validity of treatments and the best interests of patients. In Japan, it has been previously reported that decisions regarding patient treatment are often made by physicians alone (Yaguchi et al. 2005). However, our study found that many ICUs held multidisciplinary team meetings to decide on treatment policies, rather than physicians alone making such decisions during normal times before the pandemic. Furthermore, it was also shown that the multidisciplinary approach established during normal times was maintained even during the pandemic. However, 26.2% of respondents experienced ethical and social issues during the decision-making process with other HCPs more than normal times during the pandemic (Fig. 2). This means that it is not enough to maintain the multidisciplinary approach in order to make better decisions, but that it is necessary to actively attempt to understand the intentions of patients and their families and to have discussions with other HCPs even in situations with limited resources and time.

Communication among patients, their families, and HCPs is essential for patient-centered decision-making, but during the pandemic, it was difficult to provide the medical care that patients and their families hoped for as a result of insufficient communication. As described in the Nursing Guidelines of the JSICM (Japanese Society of Intensive Care Medicine 2020; Japan Geriatrics Society 2020), to maintain communication under visitation restrictions, remote visits using telephones, electronic media, and information and communication technology should be considered alternatives. Some respondents experienced moral distress because the hospital did not give approval for such remote alternatives (Table 3). This indicates that the employment of new technology sometimes requires organizational and policy initiatives.

The United Nations has also mentioned the importance of mental health support for affected individuals and their families during the COVID-19 pandemic (United Nations 2020). In addition to the usual support provided by physicians and nurses to patients and their families, highly specialized psychological support by psychiatrists may lead to improved communication quality.

Communication between the patients and their families was also impaired. Owing to the rapid deterioration of patients’ conditions caused by COVID-19, there was sometimes not enough time to confirm a patient’s intentions. Further, the patients and their families were unable to spend the end-of-life period together because of visitation restrictions. The importance of advance care planning (ACP) and end-of-life discussions has been emphasized previously in the context of COVID-19 (Kim and Grady 2020; Curtis et al. 2020; Japan Geriatrics Society 2020). Even though communication is often limited due to the COVID-19 pandemic, engaging in such processes would make treatment and care more preferable for patients (Kim and Grady 2020).

Efforts such as strengthening the quantity and quality of communication and having ACP discussions are always important in the ICU. However, we consider that the necessity for these has become even more obvious during the COVID-19 pandemic. We believe that regular efforts to improve communication would be beneficial for pandemic conditions.

#### Conflicts Experienced by Health Care Providers

During the COVID-19 pandemic, there were situations in which it was necessary to reduce contact with patients to prevent the spread of infection, deepen patient sedation more than necessary, and refrain from certain kinds of medical care such as rehabilitation (Nacoti et al. 2020). Furthermore, conflicts also occurred owing to differences in positions and roles among workers, and as a result, unusual additional mental stress was experienced. In addition, regarding infection prevention, staff members also had to avoid close contact, leaving few opportunities for them to express their thoughts and feelings. We speculate that these conflicts led to additional mental stress and moral distress among HCPs during the COVID-19 pandemic. Hence, supporting the mental health of these individuals is a critical part of the public health response (Walton et al. 2020) because it could lead to the prevention of burnout.

#### Discrimination Experienced by Health Care Providers During the COVID-19 Pandemic

This study found that 38.1% of HCPs who participated in the study faced social discrimination. It was also found that, even in the hospital, there was a sense of discrimination and inequality between those who were providing COVID-19 medical treatment and those who were not. Social discrimination has also been reported elsewhere in Japan (Japanese Association for Disaster Medicine 2020; Japanese Government New Coronavirus Infectious Disease Control Subcommittee 2020). Although efforts to increase understanding for HCPs and infected persons, as well as to eliminate discrimination, are being made primarily by the National Infectious Disease Control Subcommittee (Japanese Government New Coronavirus Infectious Disease Control Subcommittee 2020), further measures are needed to deal with the issues.

#### Allocation of Medical Resources

Although Japan had fewer patients with COVID-19 than many other countries (Watanabe 2020), and 70% of respondents said that their institutions had prepared in advance for the allocation of medical resources, in actual fact, medical resources turned out to be insufficient in the early stages of the pandemic in Japan. Regarding the kinds of medical resources that were in shortage, 70% of the respondents indicated that PPE was lacking. Despite the importance of securing PPE to protect the safety of HCPs, there was a worldwide shortage of PPE (Ranney et al. 2020; Kleinpell et al. 2020). HCPs were extremely concerned for their own health as well as that of their families (Kleinpell et al. 2020). In the free-text responses of this survey, respondents also described situations where they were not only concerned about the health of themselves and their family members, but they also felt a conflict between ensuring the safety of their staff and accepting COVID-19 patients. The lack of PPE was thought to have caused additional mental stress to the HCPs in the early stages of the pandemic in Japan. At present in Japan, the shortage of PPE is improving, but the situation is changing as there are concerns about the shortage of medical resources such as inpatient beds and ventilators.

In fact, 4.7% of the respondents reported that they were not able to provide necessary treatment due to a lack of medical resources. Further, nearly 50% of the respondents took various measures to cope with the shortage of medical resources, such as trying to increase medical resources, transferring to another facility, and receiving support. It is clear that many of them were just barely able to cope with the shortage of medical resources, suggesting that a potential problem was occurring. After this survey was conducted, due to the rapid increase in the number of patients with COVID-19, the healthcare delivery system has collapsed in some areas, creating a situation in which critically ill patients cannot be admitted to the ICU (Sasagawa and Kobayashi 2021). At present, the allocation of medical resources is becoming a more serious issue, and it is highly likely that ethical and social issues will emerge as a result. While there is a global discussion on the allocation of limited medical resources such as ventilators and ICU beds (Mannelli 2020; Truog et al. 2020; Emanuel et al. 2020; Solnica et al. 2020), it has not been sufficiently discussed in Japan. During pandemics, the burden of choosing who is eligible for invasive treatments such as ventilators should not be concentrated on the frontline HCPs, so there is an immediate need to have a discussion concerning allocation in Japan.

### Limitations and Future Challenges

This survey shows the status of members of JSICM who are engaged in COVID-19 treatment during the period of the survey. In Japan, HCPs who are not members of JSICM are also working in ICUs, especially physicians from other departments and nurses are often not members. A limitation of this study is that it may not reflect the situation of HCPs who are not members of the JSICM.

Although we received many opinions in the free-text responses, it was difficult to conduct a detailed qualitative analysis. This was because the information in the questionnaire alone was limited in its ability to capture the context and intent of the statements regarding the episodes and perceptions experienced by each individual.

Further qualitative research is needed to pursue how HCPs have responded to the ethical and social issues that arose in Japanese ICUs during the pandemic. In addition, the situation related to the COVID-19 pandemic in Japan has changed dramatically. Therefore, the ethical and social issues faced by HCPs in the ICU are continually changing, and further research is needed to capture these changes.

## Conclusions

This study provided insight into the ethical and social issues encountered in Japanese ICUs during the COVID-19 pandemic. The ethical and social issues that we identified during the COVID-19 pandemic were mainly caused by the difficulties in communication between patients and their families owing to visitation restrictions and enhanced infection control measures. In addition, it was found that moral distress was caused by having to provide unusual treatment and care to patients, as well as by conflicts arising between HCPs.

It is important to address the ethical and social issues identified in this study not only as individuals but also as medical organizations and society as a whole. To compensate for the limited contact with the patient’s family, it may be effective to make use of virtual methods such as online meetings with supports by HCPs. As psychological support for medical personnel, it is important to create a system to regularly check the mental stress of HCPs and to proactively intervene with psychologists and psychiatrists. Furthermore, providing support by professionals of medical and clinical ethics to address the issues may also be helpful. These measures could help lead to the provision of appropriate medical care for patients and their families even in difficult situations where there are various restrictions due to COVID-19 or other pandemics.

It is essential to understand the problems that cause the moral distress felt by HCPs and to take measures to alleviate them, which will help to improve working conditions and prevent burnout. We hope that the findings of this study and the future responses to these issues will lead to improvements in the medical care provided by Japanese ICUs in normal times as well as during pandemics.

## Supplementary Information

Below is the link to the electronic supplementary material.

Supplementary file1 (PDF 320 KB)

## Data Availability

All data generated or analyzed during this study are included in this published article and its supplementary information files.
