# Supplementary material for: Ethical and Social Issues for Health Care Providers in the Intensive Care Unit during the Early Stages of the COVID-19 Pandemic in Japan: a Questionnaire Survey
Source: Asian Bioeth Rev. 2021 Nov 15;14(2):115–31. doi: 10.1007/s41649-021-00194-y (PMC8590925; doi:10.1007/s41649-021-00194-y)

Ethical and social issues for healthcare providers in the intensive care unit during the early stages of the COVID-19 pandemic in Japan: A questionnaire survey

Yusuke Seino, Yayoi Aizawa, Atsushi Kogetsu, Kazuto Kato

Department of Biomedical Ethics and Public Policy, Graduate School of Medicine, Osaka University, Osaka, Japan

Corresponding author: Kazuto Kato, PhD

Department of Biomedical Ethics and Public Policy, Graduate School of Medicine, Osaka University

2-2 Yamadaoka, Suita, Osaka 5650871, Japan

Phone: +81 6 6879 3687

Email: kato@eth.med.osaka-u.ac.jp

Additional file 1 Table S1. Questionnaire items

Q1: What is your profession?

1) Physicians 2) Nurses 3) Clinical engineers 4) Physical and occupational therapists 5) Pharmacist 6) Other

Q2: How long have you been working?

1) 1–2 years 2) 3–5 years 3) 6–10 years 4) 11–20 years 5) More than 21 years

Q3: What is your work experience in the intensive care unit?

1) Currently working full-time in the intensive care unit

2) Currently working concurrently in the intensive care unit

3) Worked in the intensive care unit in the past and currently indirectly in intensive care unit work

4) Worked in the intensive care unit in the past and not currently involved in intensive care unit work

5) Has not worked in the intensive care unit in the past, but currently involved indirectly in intensive care unit work

6) Has not worked in the intensive care unit in the past and no current involvement in intensive care unit work

Q4: How many years of experience do you have in the intensive care unit?

1) 1–2 years 2) 3–5 years 3) 6–10 years 4) 11–20 years 5) More than 21years 6) No experience

Q5: What is the type of your hospital?

1) University hospital 2) Public hospital 3) Other

Q6: How was your intensive care unit operated before the COVID-19 epidemic?

1) Open intensive care unit†

2) Semi-closed intensive care unit††

3) Closed intensive care unit†††

† Intensivists are the patient's primary attending physician.

†† Mandatory critical care consultation (intensivists are not the patient's primary attending physician, but every

patient admitted to the intensive care unit receives a critical care consultation) or elective critical care consultation (intensivists are involved in the care of the patient only when the attending physician requests a consultation.)

††† No critical care physician (intensivists are unavailable).

Q7: Does your hospital belong to a designated medical institution for infectious diseases (Specified, Class 1 and Class 2 infectious diseases prescribed in the Act on Prevention of Infectious Disease and Medical Care of Infectious Patients designated infectious disease)?

1) Yes 2) No

Q8: Does your hospital have a mechanism for examining clinical ethical issues? (multiple choice)?

1) An independent hospital ethics committee

2) Other ethics committees

3) An ethics consultation system

4) No mechanism for considering clinical ethical issues

Q9: What is the most common method of making decisions on clinical ethical issues among healthcare providers in your hospital?

1) Only physician in charge

2) Physicians in a single clinical department

3) Physicians in multiple clinical departments

4) Physician and nurse in a single clinical department

5) Multidisciplinary meeting

6) Third parties, such as ethics consultation and ethics committee

7) Other

Q10: What do you recognize as ethical and social issues related to providing medical care in the intensive care unit? (Multiple choice)

1) Decision-making process among healthcare providers

2) Decision-making process with the patients

3) Decision-making process with the patient’s family

4) Limitations of life-sustaining treatment

5) Lack of palliative care and mental support for the patients

6) Lack of palliative care and mental support for the patient's family

7) Inadequate mental support for healthcare providers

8) Other

9) No problems

Q11: What do you particularly recognize as ethical and social issues related to providing medical care in the intensive care unit? (Free description)

Q12: Do you usually feel moral distress regarding medical treatment and care in the intensive care unit?

1)Yes 2) No

Q13: What do you specifically recognize as moral distress? (Free description)

Q14: Does your hospital treat confirmed or suspected cases of COVID-19? Or have you done so in the past?

1) Yes 2) No

Q15: What kind of changes did the medical care system in your intensive care unit have due to the COVID-19 pandemic? (multiple choice)

1) Increased the number of staff on duty

2) Decreased the number of staff on duty

3) Increased the amount of time to work

4) Decreased the amount of time to work

5) Received support for staff from other hospitals and departments

6) Limiting the number of available ICU beds

7) Increased the number of available ICU beds

8) Decreased the number of ICU inpatients

9) Increased the number of ICU inpatients

10) Restricted scheduled surgery

11) Changed of ICU admission/discharge criteria

12) Strengthen restrictions on family visits

13) Enhancing infection control measures

14) Limited regular conferences and study meetings

15) Other

16) No effect

Q16: Did your hospital prepare for a support system for ethical issues as part of the preparation for COVID-19?

1)Yes 2) No

Q17: What kind of preparation did your hospital prepare for the support system for ethical issues as part of the preparation for COVID-19? (Free description)

Q18: What is the most common method of making decisions on clinical ethical issues among healthcare providers in your hospital during the COVID-19 pandemic?

1) Only physician in charge

2) Physicians in a single clinical department

3) Physicians in multiple clinical departments

4) Physician and nurse in a single clinical department

5) Multidisciplinary meeting

6) Third parties, such as ethics consultation and ethics committee

7) Other

Q19: This is a question for those who answered that the methods of making decisions in normal time (Q9) and during the COVID-19 pandemic (Q18) were different. Why were the methods of making a decision different? (Multiple choice)

1) Shortage of healthcare providers

2) Increase in the number of patients

3) Decrease in the number of patients

4) Rapid deterioration of the medical condition of COVID-19 patients

5) Insufficient understanding of the medical condition

6) Lack of communication due to infection control such as visit restrictions

7) Facility policy

8) Social impact of COVID-19

9) Target of clinical trials/research

10) Other

Q20: What do you feel was not enough to support patients and their families when making decisions in situations of clinical ethical issues during the COVID-19 pandemic? (Multiple choice）

1) Providing appropriate information to both the patient and their family

2) Sharing the thoughts of patients and their family

3) Respecting the patient's will

4) Palliative care and mental support for the patients

5) Palliative care and mental support for the patient’s family

6) Other

7) Support both to patients and their families in the same way as usual

Q21: Why did you feel that you did not have enough support for the patients and their families? (Free description)

Q22: What do you feel were the ethical and social issues that healthcare providers recognized in providing medical care more than normal times during the COVID-19 pandemic? (Multiple choice)

1) Decision-making process among healthcare providers

2) Decision-making process with the patients

3) Decision-making process with the patient’s family

4) Limitation of life-sustaining treatment restrictions

5) Lack of palliative care and mental support for the patients

6) Lack of palliative care and mental support for the patient’s family

7) Inadequate mental support for healthcare providers

8) Other

9) No problems

Q23: What do you particularly recognize as ethical and social issues related to providing medical care in the intensive care unit during the COVID-19 pandemic? (Free description)

Q24: Do you feel unusual moral distress regarding medical treatment and care in the intensive care unit during the COVID-19 pandemic?

1)Yes 2) No

Q25: What do you specifically feel about unusual moral distress during the COVID-19 pandemic? (Free description)

Q26: Did your hospital prepare in advance for the allocation of medical resources necessary for life support?

1) Affiliated facilities prepared in-facility guidelines for medical resource allocation

2) Discussed the necessary medical resource allocation beforehand

3) No preparation was done.

4) Other

Q27: Did your hospital have a problem with the allocation of medical resources necessary for life support?

1) Shortage of medical resources (to the extent that the necessary treatment and care could not be provided)

2) Although medical resources seemed to be in short supply, it was possible to request support such as transfer from another facility or government.

3) Although medical resources seemed to be in short supply, it was possible to deal with it by increasing medical resources.

4) Although medical resources seemed to be in short supply, it was possible to utilize existing medical resources.

5) No problem

6) Other

Q28: Which medical resources were in shortage? (Multiple choice)

1) Human resources (physician)

2) Human resources (nurse)

3) Human resources (other staff)

4) Ventilator

5) ECMO (Extracorporeal membrane oxygenation)

6) ICU bed

7) Insufficient personal protective equipment that requires medical treatment restrictions

8) Other

Q29: Who made the decision when allocating medical resources necessary for life support? (Multiple choice)

1) Individual

2) Few healthcare providers

3) Multidisciplinary conference

4) The hospital director

5) Ethics consultation or ethics committee

6) A department specializing in the triage of medical resources

7) The ICT (infection control team)

8) Other

Q30: What did you feel was the problem when you distributed medical resources at your hospital? (Free description)

Q31: To those who answered in Q27 that the allocation of medical resources necessary for life support was not a problem. If life-sustaining medical resource allocation becomes an issue in the future,

Who would make the decision when allocating medical resources necessary for life support? (Multiple choice)

1) Individual

2) Few healthcare providers

3) Multidisciplinary conference

4) The hospital director

5) Ethics consultation or ethics committee

6) A department specializing in the triage of medical resources

7) The ICT (infection control team)

8) Other

Q32: To those who answered in Q27 that the allocation of medical resources necessary for life support was not a problem. What did you feel was the problem when you distributed medical resources at your hospital if life-sustaining medical resource allocation becomes an issue in the future? (Free description)

Q33: Did you feel prejudice or discrimination from society as a healthcare provider during the COVID-19 pandemic?

1) I did not feel it.

2) I did not feel it, but I sometimes hesitated to interact with others directly.

3) I had experienced an episode of prejudice or discrimination. but it did not become a mental burden.

4) I had experienced an episode of prejudice or discrimination, which was a mental burden.

5) Other

Q34: Please give us your opinion on ethical and social issues in the ICU during the COVID-19 pandemic. (Free description)

Table S2. Method of making decisions on clinical ethical issues among healthcare providers (N = 189)

	Normal	During the coronavirus disease (COVID-19) pandemic
Only physician in charge	14 (7.4%)	15 (7.9%)
Physicians in a single clinical department	24 (12.7%)	21 (11.1%)
Physicians in multiple clinical departments	24 (12.7%)	25 (13.2%)
Physician and nurse in a single clinical department	19 (10.1%)	17 (9.0%)
Multidisciplinary meeting	90 (47.6%)	95 (50.3%)
Third parties, such as ethics consultation and ethics committee	14 (7.4%)	10 (5.3%)
Other	4 (2.1%)	6 (3.2%)

Fig S1. Ethical and social issues in providing medical treatment and care in the ICU during normal times (multiple-choice question)

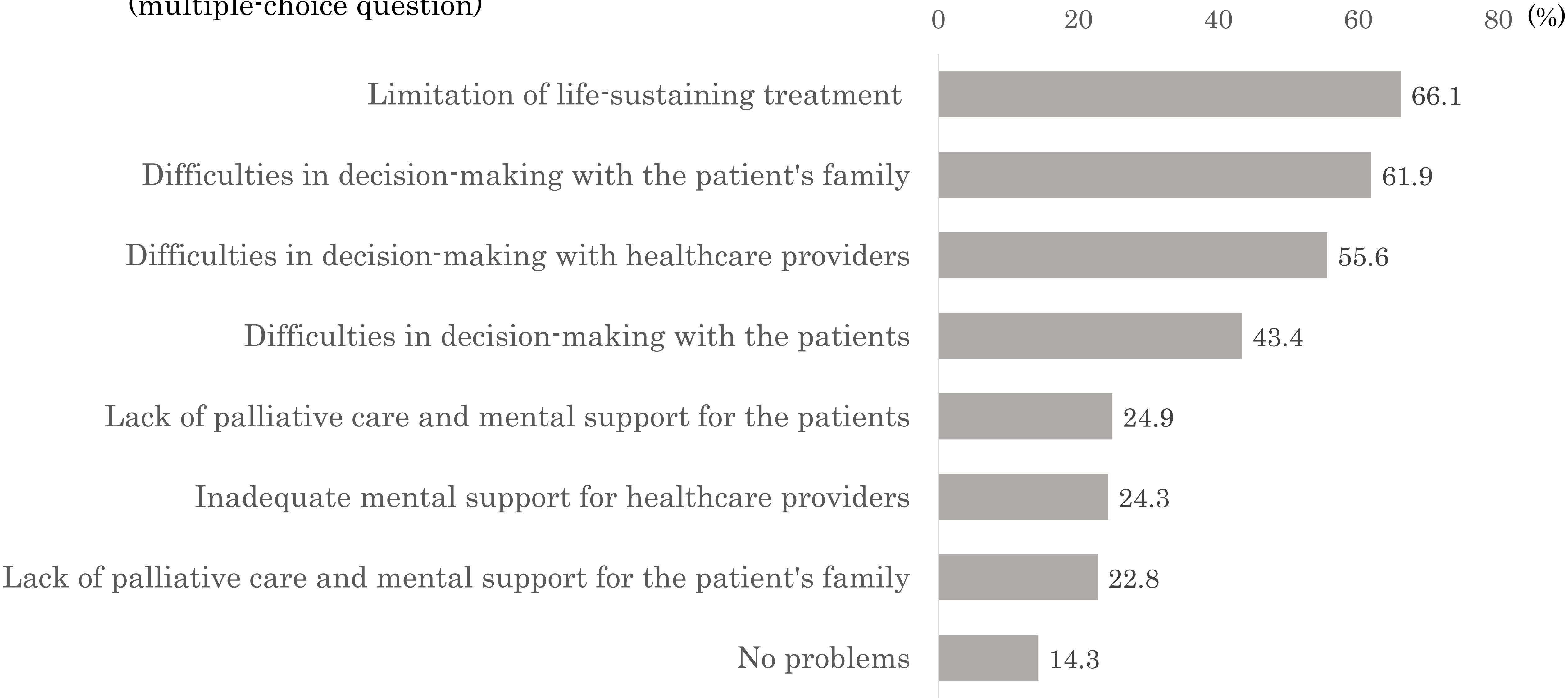

Fig S2. Points on which healthcare providers felt that they could not provide sufficient support for patients and their families (multiple-choice questions).

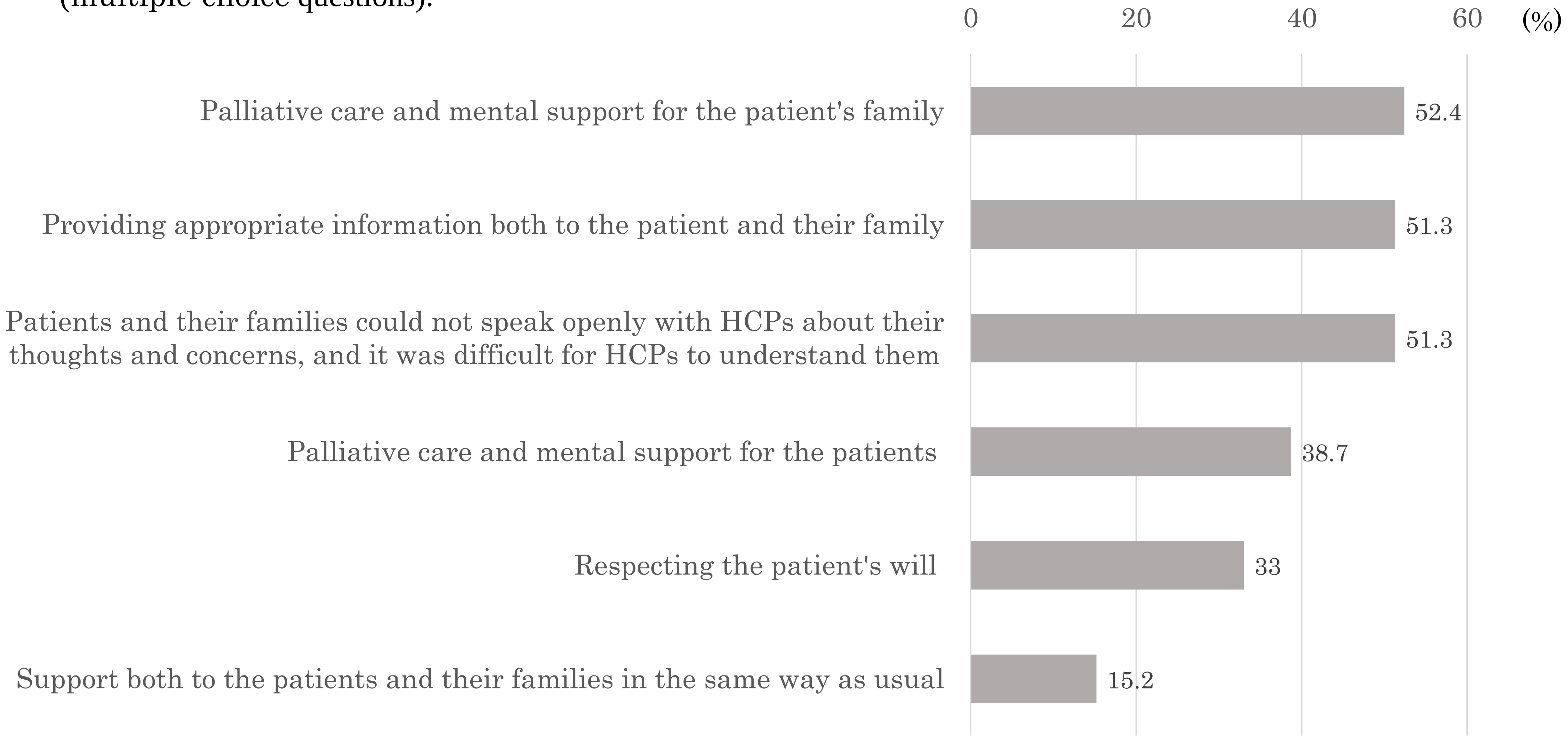

Supplement: Supplementary file 1 — Supplementary file1 (PDF 320 KB) [file 41649_2021_194_MOESM1_ESM.pdf]
